# Being a front-line dentist during the Covid-19 pandemic: a literature review

**DOI:** 10.1186/s40902-020-00256-5

**Published:** 2020-04-24

**Authors:** Hamid Reza Fallahi, Seied Omid Keyhan, Dana Zandian, Seong-Gon Kim, Behzad Cheshmi

**Affiliations:** 1 School of Advanced Technologies in Medicine, Shahid Beheshti University of Medical Sciences, Tehran, Iran; 2 Dental Research Center, Research Institute of Dental Sciences, Shahid Beheshti University of Medical Sciences, Tehran, Iran; 3 CMFRC, National Advance Center for Craniomaxillofacial Reconstruction, Tehran, Iran; 4 Craniomaxillofacial Research Center, Tehran University of Medical Sciences, Tehran, Iran; 5 Bahar building, Shahid Nuranian Alley, Saadat Abad, Isfahan, Iran; 6 Department of Oral and Maxillofacial Surgery, College of Dentistry, Gangneung-Wonju National University, Gangneung, 28644 Republic of Korea; 7 Department of Oral and Maxillofacial Surgery, College of Dentistry, Gangneung-Wonju National University, 7 Jukhyun-gil, Gangneung, 25457 Republic of Korea; 8 Faculty of Dentistry, Boroujerd Islamic Azad University, Boroujerd, Iran

**Keywords:** Coronavirus, Covid-19, 2019-nCoV, Dental care, Dentistry

## Abstract

Coronavirus is an enveloped virus with positive-sense single-stranded RNA. Coronavirus infection in humans mainly affects the upper respiratory tract and to a lesser extent the gastrointestinal tract. Clinical symptoms of coronavirus infections can range from relatively mild (similar to the common cold) to severe (bronchitis, pneumonia, and renal involvement). The disease caused by the 2019 novel coronavirus (2019-nCoV) was called Covid-19 by the World Health Organization in February 2020. Face-to-face communication and consistent exposure to body fluids such as blood and saliva predispose dental care workers at serious risk for 2019-nCoV infection. As demonstrated by the recent coronavirus outbreak, information is not enough. During dental practice, blood and saliva can be scattered. Accordingly, dental practice can be a potential risk for dental staff, and there is a high risk of cross-infection. This article addresses all information collected to date on the virus, in accordance with the guidelines of international health care institutions, and provides a comprehensive protocol for managing possible exposure to patients or those suspected of having coronavirus.

## Background

Since the first reported case in Wuhan, China, in December 2019, coronavirus disease-19 (Covid-19) has widely spread to Japan, Korea, Iran, and many European countries [1]. The World Health Organization (WHO) declared a pandemic in March 2020. As saliva is a main tool of spread, dentists are in danger of contracting Covid-19. Although the exact nature of this disease must be clarified in detailed studies, current knowledge of coronavirus infection should be shared without any restrictions.

This article was written by an Iranian team of oral and maxillofacial surgeons. As Iran has many Covid-19 patients, they have significant experience with this disease. *Maxillofacial Plastic and Reconstructive Surgery* is an open-access journal, and this type of important information can be shared via our publication platform without restrictions.

## Coronaviruses

Coronaviruses are enveloped viruses with a positive-sense single-stranded RNA genome. Their helical symmetry nucleocapsid is approximately 26–32 kb in size, making it the largest investigated genome among RNA viruses [2, 3]. Coronaviruses have a fundamental resemblance in their organization and genome expression [4]. Previously, it was thought that coronaviruses only cause enzootic infections in a number of animals, including certain birds and mammals, but recent findings indicate that a variety of these viruses, including antigenic groups 1 (229E and NL63), antigenic groups 2 (OC43), and HKU1, can infect humans [5, 6]. These viruses often lead to upper respiratory tract infection, frequently resulting in common cold symptoms. Three specific strains of these viruses that are of zoonotic origin, including severe acute respiratory syndrome coronavirus (SARS-CoV), Middle East respiratory syndrome coronavirus (MERS-CoV), and 2019 novel coronavirus (2019-nCoV), have recently caused lethal infections in humans [4, 6, 7].

Coronavirus infections in humans mainly affect the upper respiratory tract and to a lesser extent the gastrointestinal tract. Manifestations of coronavirus infections can range from relatively mild (similar to the common cold) to severe (bronchitis, pneumonia, and renal involvement) [8] (Table 1).

**Table 1:** Comparison of clinical symptoms and incubation time of human coronaviruses [4]

Human coronaviruses	Clinical symptoms	Incubation period	Refs.
229E	General malaise, headache, nasal discharge, sneezing, sore throat, fever and cough (10–20% of patients)	2–5 days	[9–11]
OC43	General malaise, headache, nasal discharge, sneezing, sore throat, fever and cough (10–20% of patients)	2–5 days	[9, 11–13]
NL63	Cough, rhinorrhea, tachypnea, fever, hypoxia, obstructive laryngitis (croup)	2–4 days	[14–19]
HKU1	Fever, running nose, cough, dyspnea	2–4 days	[20–22]
SARS-CoV	Fever, myalgia, headache, malaise, chills, non-productive cough, dyspnea, respiratory distress, diarrhea (30–40% of patients)	2–11 days	[14–19]
MERS-CoV	Fever, cough, chills, sore throat, myalgia, arthralgia, dyspnea, pneumonia, diarrhea and vomiting (one third of patients), acute renal impairment	2–13 days	[15, 23, 24]
2019-nCoV	Malaise, fever, dry cough, cough, dyspnea, myalgia, fatigue	1–14 days	[25–28]

## Pathogenesis

The ability to infect humans is mainly due to the infection of peridomestic animals, which are considered intermediate hosts, nurturing recombination and mutation events as well as the development of genetic diversity among coronaviruses [29]. Studies have suggested that the spike glycoprotein (S glycoprotein) plays an important role in host range restriction by attaching virions to the host cell membrane [30]. Generally, coronaviruses primarily replicate in the respiratory and intestinal epithelial cells and subsequently cause cytopathic alterations [31].

## Covid-19

Since December 2019, numerous unexplained cases of pneumonia have been reported in China. The disease caused by 2019-nCoV was called Covid-19 by the WHO in February 2020 [32]. Limiting the exposure of suspicious cases to the rest of society could be an effective strategy in the early outbreak phases. However, the subsequent worldwide virus spread and person-to-person transmission made the situation more complex and uncontrollable [33].

No detailed studies have been conducted to expound the pathogenicity of 2019-nCoV on a molecular scale. However, exploratory data established via whole-genome sequencing and subsequent bioinformatics analyses revealed that 2019-nCoV is phylogenetically related to SARS-CoV that was isolated for the first time in Chinese horseshoe bats between 2015 and 2017 [34–36].

### Clinical manifestations

**Fig. 1:**
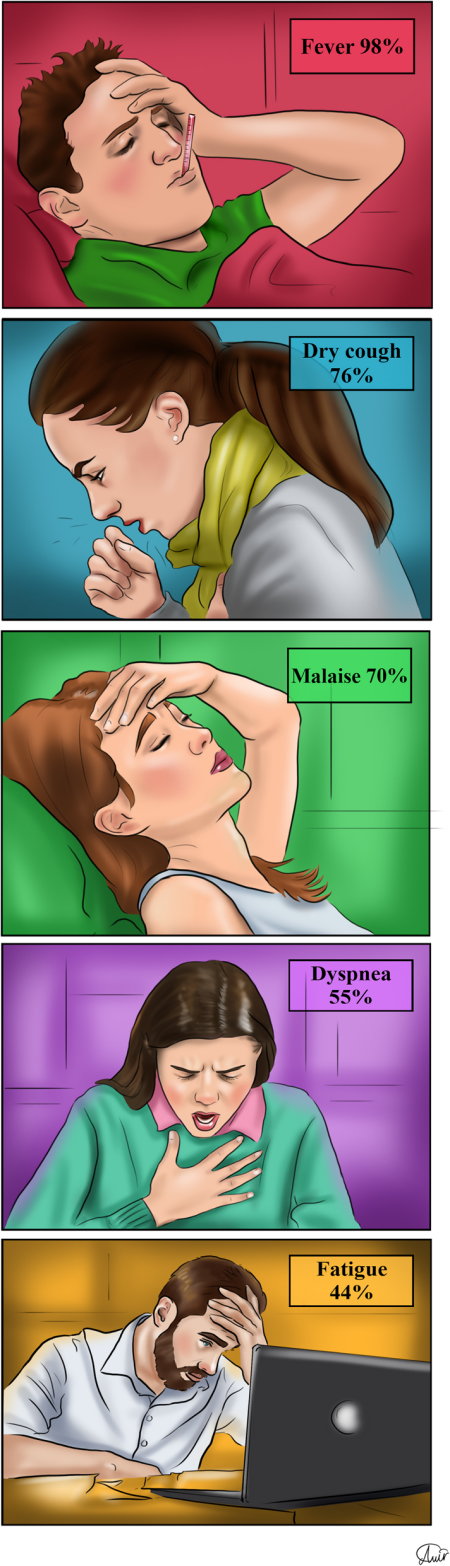
Signs and symptoms of Covid-19

To a large extent, the clinical similarities of 2019-nCoV infection with SARS-CoV infection are substantial. The incubation period of 2019-nCov has been estimated to be 1–14 days, and it has been shown that asymptomatic individuals may also be involved in the spread of this virus [26–28]. Since the possibility of the transmission from asymptomatic carriers has been raised currently, checking body temperature only may not be enough to screen asymptomatic carriers. According to a recent report, temperature-based screening in the airport can detect only 46% of 2019-nCoV carriers and the others were found during the self-isolation period after immigration [29]. To suppress the disease spread, a wide range of laboratory tests for immigrants and general population seems to be necessary. However, the infection rate from asymptomatic carriers has not been clarified until now. The primary non-specific reported symptoms of 2019-nCoV infection at the prodromal phase are malaise, fever, and dry cough. The most commonly reported signs and symptoms are fever (98%), cough (76%), dyspnea (55%), and myalgia or fatigue (44%) [25, 26]. Unlike patients with other human coronavirus infections (such as SARS-CoV), upper respiratory tract and intestinal manifestations such as sore throat, rhinorrhea, and diarrhea in those with 2019-nCoV infection are infrequent [15, 25, 26] (Fig. 1).

### Patient characteristics

The patient mean age is generally between 49 and 61 years. Studies have shown that males are more likely to have this infection [25, 26, 37]. The lack of serious illness in youngsters is a characteristic of SARS-CoV infection, which is similarly observed in 2019-nCoV infection [32]. Increased exposures to 2019-nCoV due to occupational requirements, for instance health care workers, maybe another factor contributing to the higher risk of infection.

### Diagnosis

Following the outbreak, the full 2019-nCoV genomic sequence was released in public databases [38]. This facilitates the way for further PCR assays for virus detection. The WHO recommendations for outpatient cases and patients with more critical conditions respectively include rapid collection and nucleic acid amplification testing (NAAT) of respiratory samples including nasopharyngeal and oropharyngeal swabs as well as sputum and/or endotracheal aspirate or bronchoalveolar lavage [39]. Table 2 presents recommended instructions that all practitioners in the field of dental care, including dentists, assistants, and others, should consider when treating patients or those suspected of having coronavirus.

**Table 2:** Standard precautions based on CDC and ADA guidelines for dentists on the coronavirus disease [40–42]

Postponing	Following the announcement of disease outbreak by international or local authorities, dentists can play a significant role in disrupting the transmission chain, thereby reducing the incidence of the disease by simply postponing all non-emergency dental care for all patients.
**Where to treat**	All dental care should be provided in an outpatient dental setting with a minimum of six air changes per hour, such as a hospital with dental care services or customized clinics equipped for Covid-19 patients.
**Symptoms and history**	Primary non-specific reported symptoms of 2019-nCoV infection at the prodromal phase are malaise, fever, and dry cough. The most commonly reported signs and symptoms are fever (98%), cough (76%), dyspnea (55%), and myalgia or fatigue (44%).
	They also may have traveled to one of the countries considered disease hotspots in the prior 14 days or have encountered people from those countries or people who have traveled to those countries.
	Some patients may be asymptomatic or have unexpected symptoms such as diarrhea.
**How long?**	Since it is not possible to know the etiology of each patient’s illness, it is crucial to follow the guidelines and precautions at all times during the disease outbreak.
**Preparations and arrangement**	Be alert, identify patients with respiratory illnesses, and provide them a disposable surgical face mask. Isolate them in a room with the door closed. Limit their direct contact with others. Isolated patients must wear masks outside their room.
	Isolate suspected patients before and during care to minimize their direct contact with other patients and staff and immediately report any cases to local and state public health authorities.
**Transmission prevention consideration**	To prevent 2019-nCoV transmission, dental practices should adhere to the infection control protocol, including hand hygiene, providing tissues and no-touch receptacles, and providing face masks for coughing patients.
	Dental health care personnel should wear white coats, gowns, head caps, goggles, face shields, masks, latex gloves, and impermeable shoe covers to prevent exposure.
	Disposable masks should be substituted between patients or even during treatment if they get wet.
**Guidelines updates**	Since Covid-19 recommendations may change rapidly with increasing information about the disease, the ADA recommends checking for updates on the CDC’s coronavirus infection control web page for health care professionals.
**Health care workers**	The CDC strongly recommends that all health care staff, including dentists and personnel, should receive the flu vaccine and that staff with influenza must not report to work.

## Protocol

**Fig. 2:**
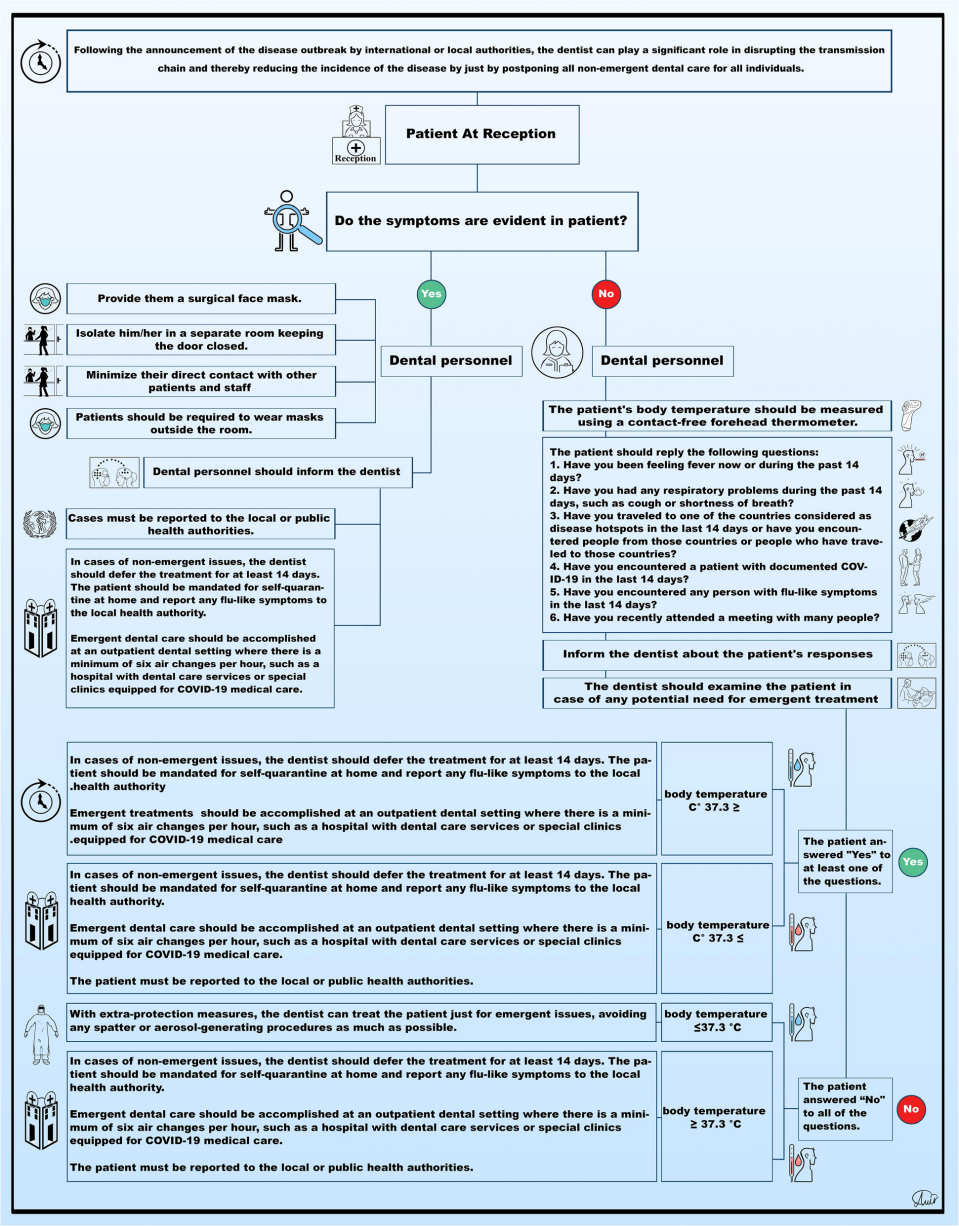
Protocol for the management of patients during the Covid-19 pandemic

Figure 2 shows a protocol that can organize our approach to managing suspected or infected patients. The purpose of this protocol is to protect the entire dental care team, prevent any cross-infection in the office, inform health authorities active in the field of controlling and managing the disease, and ultimately provide the optimal medical and dental care for patients affected by the virus according to the CDC and the ADA guidelines.

## Transmission dynamics

The two main routes known for 2019-nCoV transmission include (1) direct transmission (through coughing, sneezing, and inhalation of droplets) and (2) contact transmission (through contact with nasal, oral, and ocular mucosa) [43]. Typical clinical manifestations of Covid-2019 do not comprise ocular symptoms. However, conjunctival sample analysis has revealed that the transmission of 2019-nCoV is not limited to the respiratory tract route [26], but ocular exposure is also an effective method of virus transmission [44]. Moreover, studies have revealed that via direct/indirect contact or course and/or droplets, respiratory viruses such as 2019-nCoV may be transmitted from human to human. Studies have also shown direct and indirect transmission of 2019-nCoV through saliva [45].

**Fig. 3:**
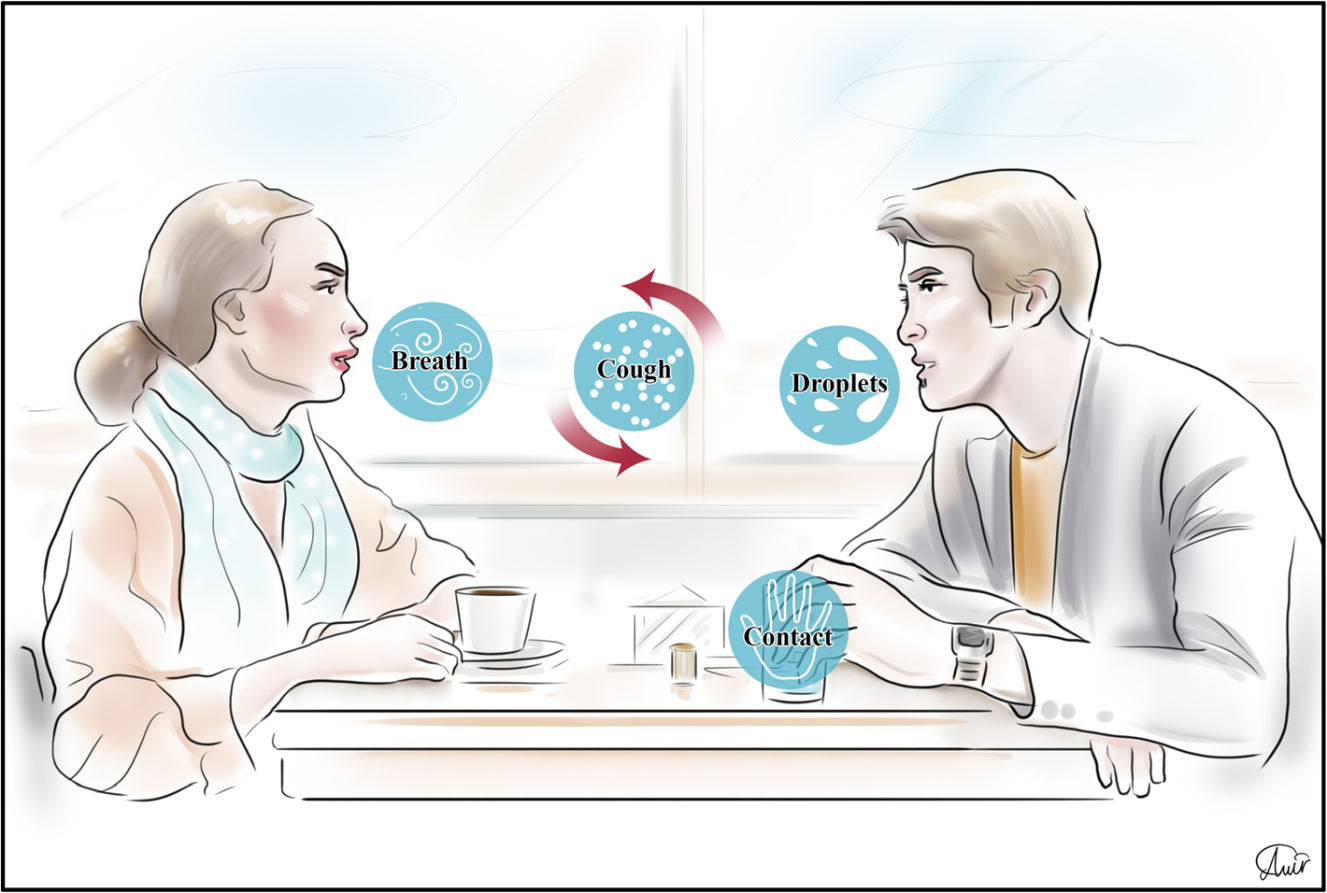
Common transmission routes of the 2019-nCoV

For a comprehensive understanding of the transmission dynamics of 2019-nCoV, it is also important to know that this virus is also transmissible through asymptomatic patients [34]. The remarkable feature of 2019-nCoV is that its RNA is detectable via quantitative reverse transcriptase polymerase chain reaction (qRT-PCR) in stool samples after the first week of infection [46]. However, the aerosol and fecal-oral transmission routes, which carry more public concern, still need further investigation and confirmation [47] (Fig. 3).

### Transmission dynamics in dentistry practice

New evidence suggests that 2019-nCoV may be transmitted directly from human to human via respiratory secretion containing droplets [44, 48]. Virus transmission through contact and fomites is also likely [44, 48]. To et al. [44] reported that using the viral culture method, they detected 2019-nCoV in infected individuals’ saliva samples. In addition, 2019-nCov invades the cells through the angiotensin-converting enzyme 2 (ACE2) receptor in the same way as the SARS coronavirus [49]. Since 2019-nCoV effectively uses ACE2 receptor for cell invasion, it can promote human-to-human transmission [50]. ACE2+ cells are abundantly present all over the respiratory tract. ACE2+ epithelial cells present in the salivary glands were considered one of the main targets of SARS coronavirus infection. Similarly, 2019-nCoV may also use the same mechanism to induce infection, although definitive judgment regarding this issue needs further study [51].

**Fig. 4:**
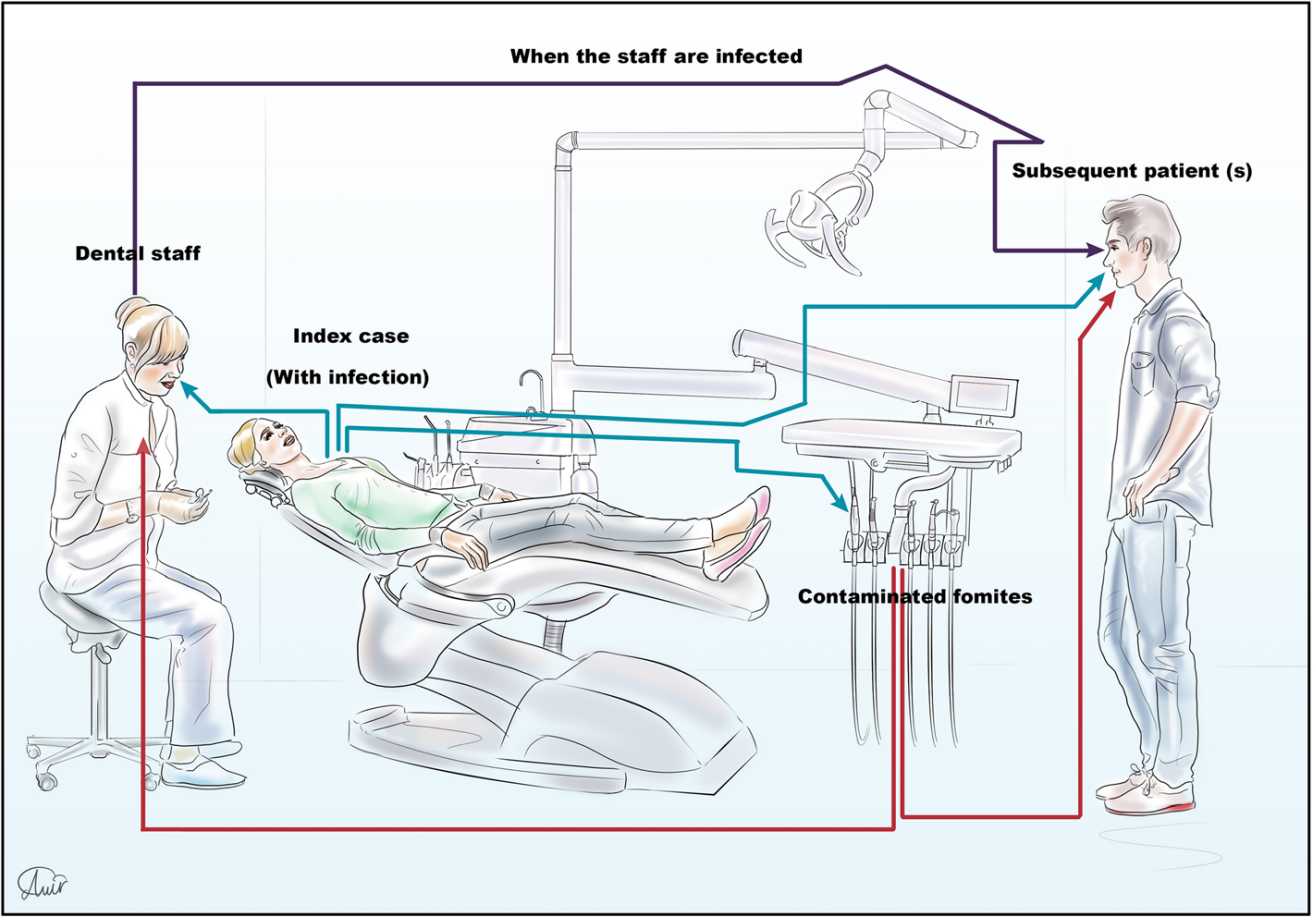
2019-nCoV transmission dynamics in dental care settings

Due to close face-to-face contact with patients and frequent utilization of sharp devices, dental personnel are repeatedly exposed to respiratory tract secretions, blood, saliva, and other contaminated body fluids and are always at risk for 2019-nCoV infection. 2019-nCoV transmission in dental settings occurs through four major routes: (1) direct exposure to respiratory secretions containing droplets, blood, saliva, or other patient materials; (2) indirect contact with contaminated surfaces and/or instruments; (3) inhalation of suspending airborne viruses; and (4) mucosal (nasal, oral, and conjunctival) contact with infection-containing droplets and aerosols that are propelled by coughing and talking without a mask [51–55] (Fig. 4).

The most important concern in dental clinics is the transmission of 2019-nCoV via droplets and aerosol because, despite all of the precautions taken, it is almost impossible to reduce droplet and aerosol production to zero during dental procedures [54]. Dental handpieces utilize high-speed gas to rotate with running water, which leads to the generation of a considerable amount of droplets and aerosol mixed with patients’ saliva and/or blood [55]. Therefore, it can be deduced that 2019-nCoV is capable of transmitting through dental practice; this transmission can be from patients to clinic staff or other patients at the clinic [56].

Research has shown that coronaviruses can remain on metal, glass, and plastic surfaces for several days [52, 57]. Therefore, as surfaces in dental clinics serve as venues for droplets and aerosol mixed with patients’ saliva and/or blood, they can effectively help spread infection. Coronaviruses can actively maintain their virulence at room temperature from 2 h up to 9 days. Their activity at 50% humidity was significantly higher than 30%. Therefore, in the dental environment, it seems that keeping surfaces clean and dry will play a significant role in preventing 2019-nCoV transmission [52].

## Infection control

Since the fecal-oral route is considered one of the 2019-nCoV transmission routes, attention to hand hygiene before, during, and after dental practice is important. Dentists should exercise extreme caution to avoid contact with their own facial mucosal surfaces including their eyes, mouth, and nose. Since transmission of airborne droplet is considered one of the main routes of infection spread, application of personal protective equipment such as masks, protective goggles, gowns, helmet, gloves, caps, face shields, and shoe covers is strongly recommended for all health care personnel.

Covid-19 patients should not be treated in a regular dental care setting without special considerations. Unexpected circumstances may occur when the dentist cannot delay treatment or refer the patient to the appropriate medical institution. Under such circumstances, special protective clothing such as hazardous materials (hazmat) suits are required. If hazmat suits are not available, white coats, gowns, head caps, protective eyewear, face shields, masks, latex gloves, and virus-proof shoe covers should be used [56].

### Mouth rinses

The effect of chlorhexidine, which is commonly used for pre-procedural mouth washing in dental practice, has not yet been demonstrated to be capable of eliminating 2019-nCoV. However, oxidative agents containing mouth rinses with 1% hydrogen peroxide or 0.2% povidone-iodine are recommended. Pre-procedural use of mouthwash, especially in cases of inability to use a rubber dam, can significantly reduce the microbial load of oral cavity fluids [58].

### Rubber dam isolation

Using rubber dams due to the creation of a barrier in the oral cavity effectively reduces the generation of droplets and aerosol mixed with patient saliva and/or blood in 1 m diameter of the surgical field by 70% [59]. Following the placement of the dam, extra high-volume suction is also required for maximum prevention of aerosol and spatter from spreading [60]. If it is not possible to use rubber dams for any reason, manual tools such as Carisolvs or hand scalers are preferable.

### Anti-retraction handpiece

Throughout the COVID-19 pandemic, the use of any dental handpieces that do not have an anti-retraction function should be avoided. For emergency treatment, anti-retraction handpieces designed with anti-retractive valves can play an effective role in preventing the diffusion and dispersion of droplets and aerosol [60, 61].

### Appropriate disinfectants

Since there is still little information available regarding 2019-nCoV, relatively similar genetic features between 2019-nCoV and SARS-CoV indicate that the novel coronavirus can be vulnerable to disinfectants such as sodium hypochlorite (1000 ppm or 0.1% for surfaces and 10,000 ppm or 1% for blood spills), 0.5% hydrogen peroxide, 62–71% ethanol, and phenolic and quaternary ammonium compounds if utilized in accordance with the manufacturer’s instructions. Studies show that other biocidal agents such as 0.05–0.2% benzalkonium chloride or 0.02% chlorhexidine digluconate probably have lower efficiency. In addition to the type of disinfectant, paying attention to other factors such as the duration of use, dilution rate, and especially the expiration time following the preparation of the solution according to the manufacturer’s instructions is also crucial.

### Management of medical waste

Prior to any inappropriate accumulation, dental office waste should be routinely transported to the institution’s temporary storage facility. Reusable tools and equipment must be properly pre-treated, cleaned, sterilized, and properly stored until the next use. Dental waste resulting from the treatment of suspected or confirmed 2019-nCoV patients is considered medically infectious waste that must be strictly disposed of in accordance with the official instructions using double-layer yellow medical waste package bags and “gooseneck” ligation.

## Conclusion

Following the announcement of the disease outbreak by international or local authorities, dentists can play a significant role in disrupting the transmission chain, thereby reducing the incidence of disease by simply postponing all non-emergency dental care for all patients. Dental professionals must be fully aware of 2019-nCoV spreading modalities, how to identify patients with this infection, and, most importantly, self-protection considerations. The effect of chlorhexidine, which is commonly used for pre-procedural mouth washing in dental practice, has not yet been demonstrated to be capable of eliminating 2019-nCoV. However, the prescription of oxidative agents containing mouth rinses such as 1% hydrogen peroxide or 0.2% povidone is recommended. A higher rate of virus exposure because of occupational commitments in health care workers is considered a key factor associated with the increased risk of infection.

## Data Availability

Not applicable.

## Abbreviations

Covid-19: Coronavirus disease-19
WHO: World Health Organization
SARS-CoV: Severe acute respiratory syndrome coronavirus
MERS-CoV: Middle East respiratory syndrome coronavirus
2019-nCoV: 2019 novel coronavirus
qRT-PCR: Quantitative reverse transcriptase polymerase chain reaction
ACE2: Angiotensin-converting enzyme 2
NAAT: Nucleic acid amplification testing
